# Depletion of neutrophil extracellular traps *in vivo *results in hypersusceptibility to polymicrobial sepsis in mice

**DOI:** 10.1186/cc11442

**Published:** 2012-07-26

**Authors:** Wei Meng, Adnana Paunel-Görgülü, Sascha Flohé, Almuth Hoffmann, Ingo Witte, Colin MacKenzie, Stephan E Baldus, Joachim Windolf, Tim T Lögters

**Affiliations:** 1 University Hospital Düsseldorf, Department of Trauma and Hand Surgery, Moorenstrasse 5, 40225 Düsseldorf, Germany; 2 University Hospital Düsseldorf, Department of Microbiology, Moorenstrasse 5, 40225 Düsseldorf, Germany; 3 University Hospital Düsseldorf, Department of Pathology, Moorenstrasse 5, 40225 Düsseldorf, Germany

## Abstract

**Introduction:**

Although the formation of neutrophil (PMN) extracellular traps (NETs) has been detected during infection and sepsis, their role *in vivo *is still unclear. This study was performed in order to evaluate the influence of NETs depletion by administration of recombinant human (rh)DNase on bacterial spreading, PMN tissue infiltration and inflammatory response in a mouse model of polymicrobial sepsis.

**Methods:**

In a prospective controlled double-armed animal trial, polymicrobial sepsis was induced by cecal ligation and puncture (CLP). After CLP, mice were treated with rhDNase or phosphate buffered saline, respectively. Survival, colony forming unit (CFU) counts in the peritoneal cavity, lung, liver and blood were determined. PMN and platelet counts, IL-6 and circulating free (cf)-DNA/NETs levels were monitored. PMN infiltration, as well as organ damage, was analyzed histologically in the lungs and liver. Capability and capacity of PMN to form NETs were determined over time.

**Results:**

cf-DNA/NETs were found to be significantly increased 6, 24, and 48 hours after CLP when compared to the levels determined in sham and naïve mice. Peak levels after 24 hours were correlated to enhanced capacity of bone marrow-derived PMN to form NETs after *ex vivo *stimulation with phorbol-12-myristate-13-acetate at the same time. rhDNase treatment of mice resulted in a significant reduction of cf-DNA/NETs levels 24 hours after CLP (*P * < 0.001). Although overall survival was not affected by rhDNase treatment, median survival after 24 hours was significantly lower when compared with the CLP group (*P *< 0.01). In mice receiving rhDNase treatment, CFU counts in the lung (*P * < 0.001) and peritoneal cavity (*P * < 0.05), as well as serum IL-6 levels (*P * < 0.001), were found to be already increased six hours after CLP. Additionally, enhanced PMN infiltration and tissue damage in the lungs and liver were found after 24 hours. In contrast, CFU counts in mice without rhDNase treatment increased later but more strongly 24 hours after CLP (*P *< 0.001). Similarly, serum IL-6 levels peaked after 24 hours (*P * < 0.01).

**Conclusions:**

This study shows, for the first time, that depletion of NETs by rhDNase administration impedes the early immune response and aggravates the pathology that follows polymicrobial sepsis *in vivo*.

## Introduction

Peritonitis and sepsis are frequent complications of critically ill patients after trauma or undergoing abdominal surgery [1-3]. Peritonitis and sepsis, frequently related to a polymicrobial infection with extracellular microorganisms from the intestinal flora, continue to cause high morbidity and mortality [1,4-6].

Neutrophils (PMN) represent the first line of innate immune defense against extracellular microorganisms [3,7-9]. Equipped with a full arsenal of antimicrobial proteins at their disposal, PMN have a well described role in phagocytic uptake and intracellular killing [10,11]. Besides their ability to eliminate microorganisms by phagocytosis, it has recently been shown that activation of PMN causes the formation of neutrophil extracellular traps (NETs) [12-15]. This novel mechanism, for the first time described by Brinkmann *et al*., consists of the release of web-like structures of DNA and proteins that bind, disarm and kill pathogens extracellularly [12].

NETs have been consistently shown to entrap and kill gram-negative and gram-positive bacteria, as well as fungi [11-14,16]. Moreover, the confinement of pathogens to a local site of infection might be an important function of NETs [16]. Nevertheless, it has been suggested that the antibacterial mechanism of NETs formation occurs at the expense of injury to the host [17]. Exposure of NETs-associated proteases and other granular proteins to the extracellular environment has been shown to damage endothelial cells *in vitro *[17-19]. In models of lipopolysaccharide (LPS) induced endotoxemia, NETs have been identified in the microcirculation of the liver in the sinusoids resulting in an impaired perfusion and increased tissue damage measured by levels of alanine aminotransferase (ALT) [17].

The latter observations leave NETs as a potentially double-edged sword. On the one hand, NETs represent an important mechanism of bacterial killing and preventing the spread of pathogens from the initial site of infection. On the other hand, NET formation may have deleterious effects on the host due to the release of high levels of potentially noxious proteins like proteases inducing damage to the adjacent tissue [11].

Although NETs have been found to be abundant at sites of infection and observed in the circulation in infection and sepsis [12,20-22], the pathophysiogical relevance of NETs for *in vivo *conditions is still under debate. DNA is the main structural component and scaffold of NETs [12]. It has been shown that brief treatment of NETs with DNase abolishes microbial killing *in vitro *[12]. Of interest, bacterial trapping of *Escherichia coli *by activated PMN was reduced by prior exposure to DNase *in vivo *[17]. Recombinant human (rh)DNase catalyses the hydrolysis of extracellular DNA. Application of rhDNase has been shown to be safe and well tolerated in the treatment of patients with systemic lupus erythematosus and cystic fibrosis. Thus, administration of rhDNase offers the possibility to investigate the pathophysiological role of NETs for polymicrobial peritonitis and sepsis *in vivo*, which has not been investigated so far. The aim of this study was to evaluate the effects of the administration of rhDNase on the inflammatory response and bacterial spreading in a murine model of polymicrobial peritonitis and sepsis caused by cecal ligation and puncture (CLP).

## Materials and methods

### Ethics statement

All animal procedures were carried out under local and national ethical guidelines and were approved by the regional ethical committee, Regional Office for Nature, Environment and Consumer Protection Nordrhein-Westfalen, Germany, with the ethical approval ID 87-51.04.2010.A112.

### Animals

Wild-type C57BL/6 mice, 7 to 11-week-old females weighing 18 to 22 grams, (animal facility of the Heinrich-Heine-University Duesseldorf (Tierversuchsanlage, TVA, Germany) were used for the experiments. All mice (n = 453) were housed under specific pathogen-free conditions and had free access to standard rodent food and water *ad libitum*.

### Cecal ligation and puncture (CLP)

Polymicrobial sepsis was induced by CLP as previously described [23]. Briefly, animals were anesthetized with an intraperitoneal (i.p.) injection of a mixture of 10 mg/kg xylazine and 100 mg/kg ketamine hydrochloride. The abdomen was gently shaved and cleaned with betadine and alcohol swabs. A 1 cm midline skin incision was made and fascia as well as the peritoneum was opened. The cecum was then delivered to a sterile operative field on the abdominal surface. A 4-0 silk suture was used to ligate approximately 50% of the cecum at its proximal aspect without occlusion of the intestinal lumen. A 21-gauge needle was used to puncture the cecum twice, and a small amount of cecum content was extruded. The cecum was then replaced into the abdominal cavity, and the incision was closed with two layers. Sham mice were treated identically except for the ligation and puncture of the gut. All mice were resuscitated by an i.p. injection of 0.5 ml sterile saline. Under these conditions, all CLP mice showed signs of severe illness within 24 hours after induction of sepsis.

### Experimental design

Mice were classified into four groups: 1) sham (n = 65); 2) sham + rhDNase (n = 20); 3) CLP (n = 207); 4) CLP + rhDNase (n = 126). In addition, naive mice (n = 35) served as controls. The i.p. application of rhDNase in mice was first described by Macanovic *et al*. who indicated that rhDNase concentrations between 0.1 and 1 µg/ml were necessary to produce detectable nuclease activity in serum [24]. Animals were treated with either 5 mg/kg rhDNase (Pulmozyme, Roche, Grenzach-Wyhlen, Germany) or 100 µl PBS containing 2 mM Ca^2+ ^at 1 hour, 4 hour, 7 hour, 10 hour, 21 hour, 24 hour, and 27 hour after CLP. Sham-operated mice without cecum perforation received the same treatment. The survival rate was monitored until day six. At 6 hours, 24 hours and 48 hours after surgery the blood of anesthetized mice was collected by cardiac puncture, after which the animals were sacrificed. The peritoneal cavity was then opened and lavaged under aseptic conditions with 2.5-ml aliquot of cold PBS. Part (100 µl) of the lavage was used for bacteria cultures. Then the peritoneal lavage fluids were centrifuged separately and the supernatant was stored at -20°C for subsequent measurement of cf-DNA and DNase concentration.

### Isolation of mouse PMN from bone marrow

Bone marrow-derived PMN were isolated as described before [25]. Briefly, the bone marrow was flushed out of the tibia and the femur using RPMI 1640 containing 2 mM glutamine supplemented with 100 U/ml penicillin, 100 µg/ml streptomycin and 10 % FCS (full medium) with a 2 ml syringe. Cells were pelleted and the remaining erythrocytes were lysed using red blood cell lysis solution (0.83% ammonium chloride, 0.1 % KHCO_3 _and 0.004 % ethylenediaminetetraacetic acid (EDTA)). PMN were purified by centrifugation for 30 minutes at 500 × g on a discontinuous Percoll gradient consisting of 55% (v/v), 68% (v/v) and 78% (v/v) Percoll (Biochrom, Berlin, Germany) in PBS. Mature PMN were recovered from the interphase between 68% and 78% Percoll. This procedure revealed the purity of vital isolated PMN at 90% as confirmed by light microscopy with Diff-Quick (Medion, Dudingen, Switzerland) staining.

### Stimulation of PMN and NETs release

Freshly isolated PMN from bone marrow were resuspended in RPMI full medium to a final concentration of 2 × 10^6^/ml. Cells were further stimulated with different concentrations of phorbol-12-myristate-13-acetate (PMA, range 0 to 100 nM) for three hours at 37°C in a humidified atmosphere containing 5% CO_2_. NETs released in the supernatant were quantified as described below. In addition, supernatants of stimulated PMN isolated from naive mice were diluted (1:5) and further incubated with 0, 0.02, 0.2, 2.0 and 10 µg/ml of rhDNase (Pulmozyme) for 30 minutes at 37°C before cf-DNA/NETs quantification.

### Quantification of cell free DNA

To quantify levels of circulating free (cf)-DNA/NETs in the different groups, the Quant-iT Pico Green dsDNA assay was used according to the manufacturer's instructions (Invitrogen GmbH, Darmstadt, Germany). In addition, free DNA released by freshly isolated PMN after PMA stimulation was determined in the culture supernatant by the same method. The fluorescence intensity reflects the amount of DNA and was measured at excitation and emission wavelengths of 485 nm and 530 nm, respectively, in a microplate reader (Victor3, PerkinElmer, Waltham, MA, USA). A standard calibration curve by means of defined calf thymus DNA (Sigma, St. Louis, MO, USA) amounts ranging from 0 to 2 µg/ml was used in all analyses.

### Quantification of desoxyribonuclease (DNase) and interleukin (IL)-6 by ELISA

Desoxyribonuclease (DNase) levels in serum and peritoneal lavage samples were measured by using ORG 590 DNase Activity Immunometric Enzyme Immunoassay for the Quantitative Determination of DNase Activity (ORGENTEC, Mainz, Germany) according to the manufacturer's instructions. Additionally, the concentration of DNase in mice sera was quantified using known concentrations of the standard provided with rhDNase1 (0.75 up to 12.5 ng/ml). IL-6 levels in the sera were determined using a commercially available IL-6 ELISA kit according to the manufacturer´s instructions (R&D Systems, Abingdon, UK). Concentrations were calculated from the standard curve constructed with recombinant murine IL-6. The lower detection limit was 16 pg/ml IL-6.

### Histology and staining procedures

Leukocyte infiltration was quantified in liver and lung sections. In order to harvest lungs and livers, mice were sacrificed 6 hours, 24 hours, and 48 hours after CLP. Tissue samples were fixed in 4% formaldehyde and embedded in paraffin according to standard procedures. Sections (3 µm) were stained with H & E for pathological examination. In addition, chloracetatesterase staining was performed for specific detection and quantification of tissue infiltration by PMN. PMN were counted in a blinded and standardized fashion by light microscopy (Axiovert 40, Zeiss, Goettingen, Germany). Briefly, a micrometer ocular (x10) was used to count PMN in 20 different visual fields of each section. The histologic grading of liver injury was based on the following parameters: infiltration of inflammatory cells, necrosis, steatosis and ballooning degeneration. For the scoring of lung damage, infiltration of inflammatory cells, vascular congestion and interstitial edema were evaluated. All parameters were evaluated by the following score scale of values: 0, absent; 1, mild; 2, moderate; and 3, severe. All histopathological evaluations were done in a blinded fashion by an independent pathologist.

### Counts of colony-forming units (CFU)

The number of colony-forming units (CFU) was determined in peripheral blood, the peritoneal cavity, as well as lung and liver homogenates. Six hours, 24 hours, and 48 hours after CLP mice were sacrificed for lung and liver removal. Lungs and livers were removed under aseptic conditions and homogenized. Bacteria from the peritoneal cavity were obtained by peritoneal lavage under aseptic conditions with 2 ml cold PBS. A volume of 30 µl of extracted peritoneal lavage was stored on ice. Peripheral blood was isolated by cardiac puncture. Subsequently, 30 µl of blood and peritoneal lavage and extracts of organs were serially diluted in PBS, plated on Columbia Agar plates with 5% sheep blood and incubated under aerobic conditions with a minimum amount of vibration at 37°C. Bacterial colonies were counted after 24 hours. Results were specified as CFU per 1 ml.

### Immunofluorescence staining of NETs

For immunofluorescence, freshly isolated PMN from the bone marrow were seeded on poly-D-lysin coated coverslips, allowed to adhere, and stimulated with 50 nM PMA for three hours at 37°C. Then PMN were incubated with 2 µg/ml and 20 µg/ml rhDNase for 30 minutes. Cells were further fixed for 12 hours with 4% paraformaldehyde (PFA) and blocked with 5% normal goat serum (NGS, Dako, Hamburg, Germany), 0.3% Triton X-100 in PBS for 30 minutes. To stain NETs, samples were incubated with a monoclonal mouse anti-myeloperoxidase antibody (1:300) and a secondary fluorescein isothiocyanate (FITC)-conjugated goat anti-mouse IgG antibody (1:200; both Dako, Hamburg, Germany). After staining of DNA with 4',6-diamidino-2-phenylindole (DAPI), specimens were mounted in Dako fluorescent mounting medium (Dako). Neutrophil-derived NET formation was visualized by immunofluorescence microscopy (Axiovert 100, Zeiss).

### Statistical analyses

Statistical analyses were performed using GraphPad Prism 5.0 (GraphPad Software, San Diega, CA, USA). Data are presented as mean ± standard error of the mean (SEM). Data obtained from multiple groups were tested using one-way analysis of variance (ANOVA) followed by Newman-Keuls post-test. Data on the bacterial load are presented as scatter plots including the median and were tested using the nonparametric Mann-Whitney U test. Survival data are presented as Kaplan-Meier plots. Times of survival were compared by use of the Gehan-Wilcoxon rank sum test; prevalences of mortality were compared using Fisher's exact test. Data were considered to be statistically significant at *P * < 0.05.

## Results

### Increased NETs release and DNase concentration after CLP

**Figure 1:**
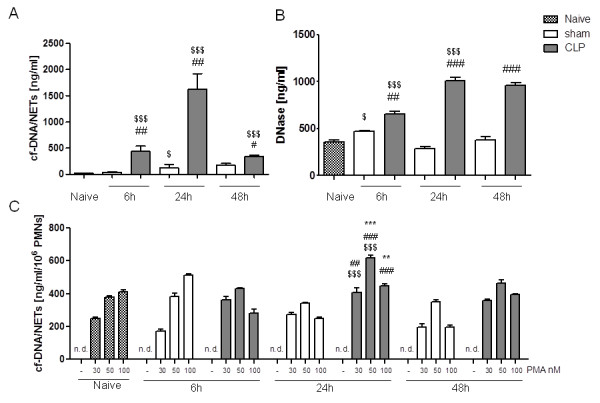
**Increased cf-DNA/NETs release and DNase concentration in peripheral blood as well as continuous capability and capacity of NETs release by PMN after CLP**. cf-DNA/NETs (**A**) and DNase (**B**) levels in serum of mice were determined 6, 24, and 48 hours after CLP or sham operation, that is laparotomy without cecum perforation. Naive mice served as controls. (**C**) Additionally, cf-DNA/NETs were quantified in the supernatants of freshly isolated PMN from naive, sham and CLP mice, respectively, and further stimulation with 0, 30, 50, and 100 nM PMA for three hours PMA **P  < 0.01, ***P  < 0.001 versus adjacent time point within one group; ^#^*P * < 0.05, ^##^*P * < 0.01, ^###^*P * < 0.001 versus sham; ^$^*P * < 0.05, ^$$$^*P * < 0.001 CLP versus naïve. Cf, circulating freely; CLP, cecal ligation and puncture; DNase, desoxyribonuclease; n.d.: not detected; PMA, phorbol-12-myristate-13-acetate; PMN, neutrophils.

Levels of cf-DNA/NETs and DNase were determined in the serum of mice at 6 hours, 24 hours and 48 hours after CLP or sham operation (Figure 1A,B). At all time points cf-DNA/NETs values were significantly increased in the CLP group compared to the sham and naive groups, with a maximum at 24 hours after CLP (*P * < 0.001; Figure 1A). Likewise, the levels of DNase in the CLP group also increased as early as 6 hours after CLP and remained elevated throughout the entire period compared to the naive and sham groups (Figure 1B). In contrast to cf-DNA/NETs levels, DNase did not decline 48 hours after sepsis induction.

### Continuous capability and capacity of NET-release after CLP

Since cf-DNA/NETs serum levels declined 48 hours after CLP a regulation or even exhaustion of this defense mechanism could be assumed. In order to verify the capacity of PMN to form NETs during a septic disease, PMN were isolated from bone marrow 6, 24, and 48 hours after CLP or sham operation and stimulated *ex vivo *with PMA. In addition, PMN from naive mice served as controls. The induced cf-DNA/NETs release was finally determined in the supernatant. PMN were able to form NETs throughout the entire period after CLP (Figure 1C). Neutrophil NET-producing capacity after *ex vivo *PMA-stimulation was significantly increased 24 hours after CLP in comparison to naive and sham-operated mice. Thus, the time kinetic of the NET-producing capacity of PMNs isolated from bone marrow reflects the kinetic of NETs concentrations in the blood stream after CLP.

### Degradation of NETs by rhDNase *in vitro *

**Figure 2:**
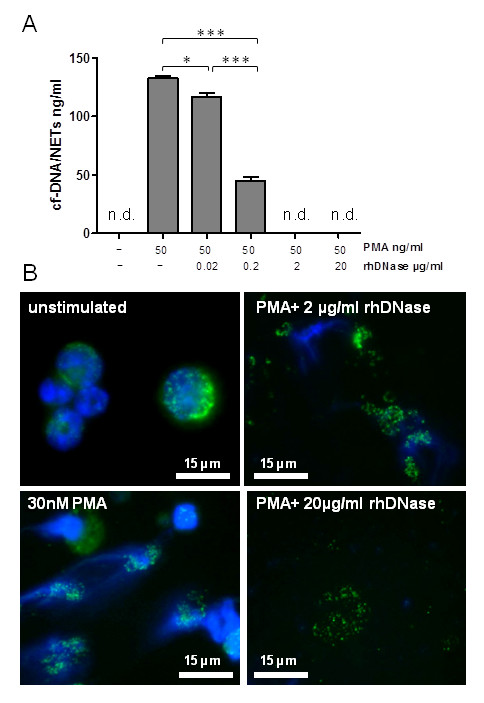
**Degradation of NETs by rhDNase *in vitro***. (**A**) PMN were isolated from bone marrow of naive mice and stimulated with 50 nM PMA. Supernatants were incubated with 0, 0.02, 0.2, 2.0 and 20.0 µg/ml rhDNase. An rhDNase concentration of 2 µg/ml resolves 140 µg/ml of NETs completely. n.d. = not detected. *P  < 0.05, ***P  < 0.001. (**B**) Immunofluorescence staining of NETs. The image of unstimulated PMN shows the nuclear localization of DNA (blue fluorescence) and the granular pattern of MPO (green fluorescence; top left). After stimulation morphological changes during NETs formation could be determined with loss of nuclear lobules and granular integrity of MPO (bottom left). Exposure of fixed NETs with 2 µg/ml (top right) and 20 µg/ml (bottom right) rhDNase resulted in the disintegration of NETs with loss of DNA structures. CLP, cecal ligation and puncture; DNase, desoxyribonuclease; MPO, myeloperoxidase; NETs, neutrophil extracellular traps; PMN, neutrophils; rh, recombinant human.

In order to prove the ability of rhDNase to degrade the scaffold of murine NETs *in vitro*, supernatants of PMA-stimulated PMN isolated from naive mice were incubated with rhDNase and NETs were quantified by a cf-DNA/NETs assay. As depicted in Figure 2A, NETs were disintegrated in a dose-dependent manner. Furthermore, qualitative evidence of rhDNase-mediated NET degradation was shown by immunofluorescence microscopy (Figure 2B). Viable unstimulated PMN and PMN stimulated with PMA were fixed and stained for NET components, that is, DNA and myeloperoxidase (MPO). Exposure of fixed NETs with rhDNase resulted in the disintegration of NETs with loss of DNA structures. Thus, in principle rhDNase is able to functionally destroy murine NETs derived by activated PMN *in vitro*.

### Degradation of NETs by rhDNase treatment *in vivo *

**Figure 3:**
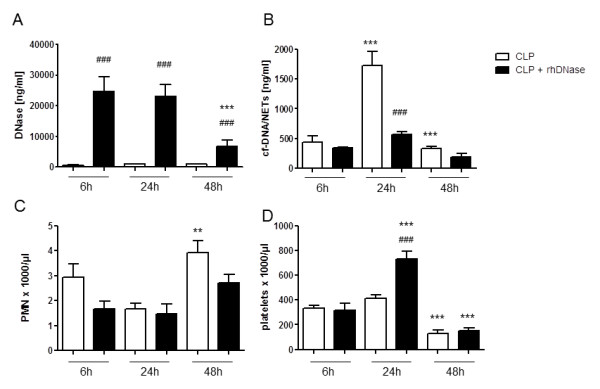
**Influence of rhDNase treatment on NETs activity, neutrophil and platelet counts**. cf-DNA/NETs (**A**) and DNase (**B**) levels as well as neutrophil (**C**) and platelet (**D**) counts in serum of mice at 6 (n = 5), 24 (n = 7), and 48 (n = 7) hours treated with either 5 mg/kg rhDNase or PBS 1, 4, 7, 10, 21, 24, and 27 hours after CLP. *P  < 0.05, **P  < 0.01, ***P  < 0.001 versus adjacent time point within one group; ^###^*P * < 0.001 versus CLP without rhDNase. CLP, cecal ligation and puncture; DNase, desoxyribonuclease; NETs, neutrophil extracellular traps; PBS, phosphate-buffered saline; rh, recombinant human

To investigate the effects of rhDNase treatment on NET activity *in vivo*, serum values of cf-DNA/NETs and DNase were determined 6, 24 and 48 hours after CLP (Figure 3). As expected, i.p. injection of rhDNase was associated with high DNase values in serum (Figure 3A). DNase values declined again 24 hours after the last rhDNase injection. However, even 48 hours after CLP, DNase serum concentrations were still elevated by almost 10,000 ng/ml. Most importantly, as a consequence of rhDNase treatment, the peak cf-DNA/NETs values in serum 24 hours after CLP was completely abolished (*P * < 0.001; Figure 3B). Thus, it has been shown that degradation of NETs by rhDNase is also possible *in vivo*.

The interaction of activated platelets with PMN has been regarded as an important NET-inducing mechanism in sepsis [15]. Therefore, neutrophil and platelet counts in peripheral blood samples after CLP have been determined (Figure 3C, D). Circulating PMN were not affected in the early phase of sepsis in terms of absolute numbers by rhDNase treatment during sepsis (Figure 3C). At 48 hours after CLP the neutrophil number was elevated significantly in comparison to the number determined after 24 hours. Interestingly, in parallel to decreased NETs 24 hours after sepsis induction in the rhDNase-treated CLP group, significantly higher amounts of platelets were found in the circulation of CLP mice treated with rhDNase at this time point (*P * < 0.001). In addition, the reduced NET production *in vivo *after 48 hours was accompanied by a platelet drop in the circulation (Figure 3D). Since platelets have been shown to be an important initiating factor for NET formation, the reduced platelet counts in septic mice after 48 hours might explain the reduced NET formation *in vivo *later during sepsis.

### Influence of NETs depletion on survival after CLP

**Figure 4:**
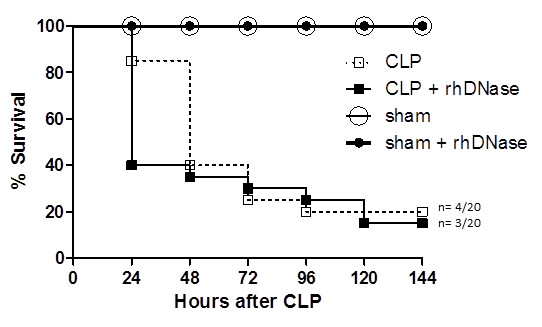
**Influence of NETs depletion on survival after CLP**. Survival curve after CLP of mice treated with rhDNase. Mice were injected intraperitoneally with either rhDNase (CLP + rhDNase, n = 20) or PBS (CLP, n = 20) 1, 4, 7, 10, 21, 24 and 27 hours after CLP and were monitored for 6 days or until death. Sham (laparotomy without CLP) groups with (n = 10) and without rhDNase (n = 6) treatment served as further controls. Mortality prevalence of mice treated with rhDNase was lower than in mice treated with PBS 24 hours after CLP (*P * < 0.01, Fisher's exact test).CLP, cecal ligation and puncture; DNase, desoxyribonuclease; NETs, neutrophil extracellular traps; PBS, phosphate-buffered saline; rh, recombinant human

For the purpose of further elucidating the relevance of NETs formation on the host-defense in polymicrobial sepsis, the influence of rhDNase treatment on survival after CLP or sham operation was analyzed. Interestingly, median survival after CLP of mice treated with rhDNase was shorter than in CLP mice (CLP + DNase: 24 hours; CLP: 48 hours), although there was no significant difference in overall survival between the two groups. However, mortality prevalence 24 hours after CLP was higher in mice treated with rhDNase than in CLP mice (*P *= 0.008, Fisher's exact test). Survival of sham mice receiving or not receiving rhDNAse did not show any differences (*P *= 0.08; Gehan-modified Wilcoxon rank sum test, Figure 4).

### Enhanced bacterial dissemination without NETs

**Figure 5:**
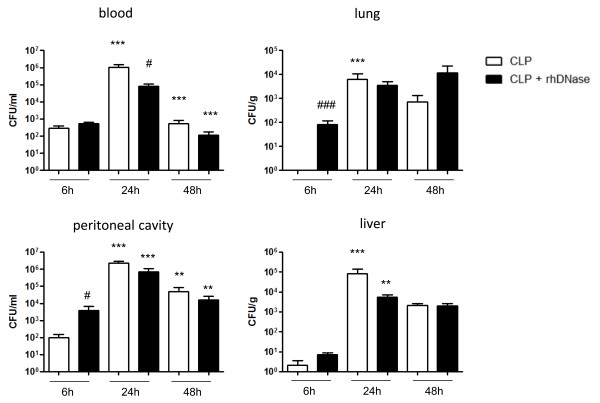
**Enhanced bacterial dissemination without NETs activity**. Mice were treated with rhDNase (CLP + rhDNase; n = 44) or PBS (CLP; n = 44), respectively. Bacterial colony forming units (CFU) were recovered 6 hours (n = 8/8 in both groups), 24 hours (n = 7/14 and n = 10/14), and 48 hours (n = 9/22 and n = 11/22) after CLP from the peritoneal cavity, peripheral blood, lung, and liver. Data from two independent experiments are depicted. **P  < 0.01, ***P  < 0.001 versus adjacent time point; ^#^*P * < 0.05, ^###^*P * < 0.001 versus CLP. CLP, cecal ligation and puncture; DNase, desoxyribonuclease; NETs, neutrophil extracellular traps; PBS, phosphate-buffered saline; rh, recombinant human.

To evaluate the effects of NETs activity on bacterial spreading during sepsis the number of CFU was determined in peripheral blood, the peritoneal cavity, lung, and liver 6, 24, and 48 hours after CLP (Figure 5). Naive or sham operated mice served as controls without any CFU counts (data not shown). As shown in Figure 5, already six hours after CLP, CFU counts were significantly enhanced in the peritoneal cavity (*P * < 0.05) and the lung (*P * < 0.001) of mice with rhDNase treatment. Interestingly, 24 hours after CLP a rebound of CFU counts in peripheral blood was visible with an increase in mice without rhDNase treatment (*P * < 0.05).

### Enhanced systemic inflammation without NETs

**Figure 6:**
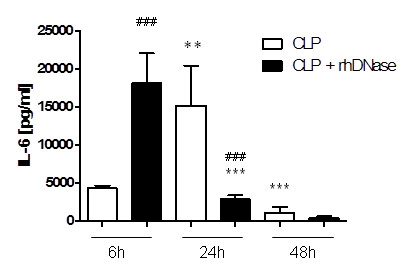
**Enhanced IL-6 levels without NETs activity**. IL-6 levels in the serum of mice 6 (n = 5), 24 (n = 7), and 48 (n = 7) hours after CLP treated with either 5 mg/kg rhDNase (CLP + rhDNase) or 100 µl PBS (CLP) 1, 4, 7, 10, 21, 24, and 27 hours after CLP. **P  < 0.01, ***P  < 0.001 versus adjacent time point; ^###^*P *< 0.001 versus CLP. CLP, cecal ligation and puncture; DNase, desoxyribonuclease; IL-6, interleukin-6; NETs, neutrophil extracellular traps; PBS, phosphate-buffered saline; rh, recombinant human.

Depletion of NETs activity may have an influence on the inflammatory response to infection. Therefore, serum levels of IL-6 were measured 6, 24, and 48 hours after CLP (Figure 6). DNase administration resulted in significantly increased IL-6 levels compared to the CLP group six hours after CLP. In contrast, IL-6 levels in the CLP mice were found to increase later with a maximum after 24 hours (*P * < 0.01). At this time, IL-6 levels were significantly decreased in mice receiving rhDNase treatment in comparison with the CLP group (*P * < 0.001, Figure 6).

### Enhanced neutrophil recruitment into affected organs without NETs

**Figure 7:**
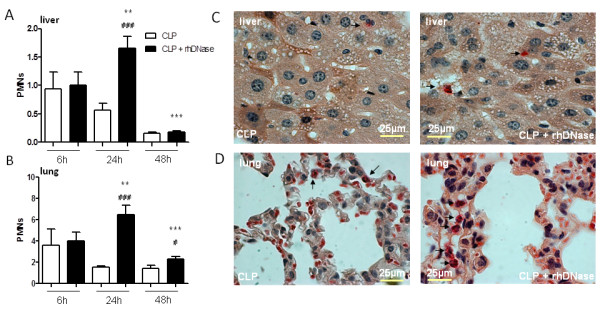
**Enhanced neutrophil recruitment into the lung and liver after rhDNase treatment**. (**A, B**) Neutrophil counts in the lung and liver of mice 6 (n = 5), 24 (n = 7), and 48 (n = 7) hours after CLP treated with either 5 mg/kg rhDNase (CLP + rhDNase) or 100 µl PBS (CLP) 1, 4, 7, 10, 21, 24, and 27 hours after CLP. **P  < 0.01, ***P  < 0.001 versus adjacent time point; ^#^*P * < 0.05; ^###^*P * < 0.001 versus CLP. Chloracetatesterase staining of paraffin sections from the lung and liver of mice 24 hours after CLP treated with either 5 mg/kg rhDNase (CLP + rhDNase) or 100 µl PBS (CLP). Representative sections of the liver (**C**) and lung (**D**) are depicted. Scale bar indicated 100 μm. CLP, cecal ligation and puncture; DNase, desoxyribonuclease; NETs, neutrophil extracellular traps; PBS, phosphate-buffered saline; rh, recombinant human.

**Figure 8:**
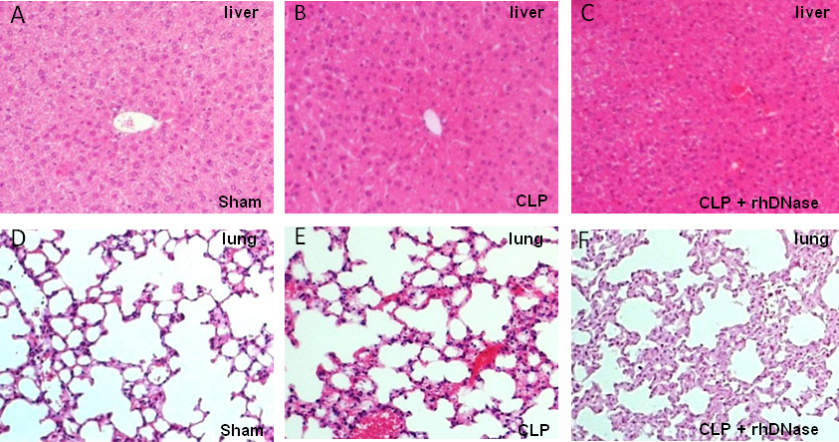
**Histology of liver and lung damage 24 hours after CLP with and without rhDNase treatment**. (**A**) Histology of lung obtained from sham mice. (**B**) Representative CLP-induced lung damage. (**C**) Representative lung of CLP mice treated with rhDNase (CLP + rhDNase). (**D**) Histology of liver obtained from sham mice. (**E**) Representative CLP-induced liver damage. (**F**) Representative liver of CLP mice treated with rhDNase (CLP + rhDNase). Sham and CLP mice were sacrificed 24 hours after surgery. Original magnification, ×10. CLP, cecal ligation and puncture; DNase, desoxyribonuclease; rh, recombinant human.

In order to analyze the effect of rhDNase treatment on neutrophil migration into sepsis-affected organs histological staining of lung and liver samples was performed. There were no differences between sham animals with or without rhDNase application (data not shown). As shown in Figure 7, disintegration of NETs by rhDNase treatment did not influence the neutrophil counts in the lung and the liver early after CLP (six hours). However, 24 hours after CLP, rhDNase treatment strongly increased the migration of PMN in the lung and liver compared to the CLP group. Forty-eight hours after CLP neutrophil counts decreased again in both groups. Representative tissue sections are depicted in Figure 7C and 7D, which show enhanced neutrophil counts as a consequence of rhDNase treatment 24 hours after CLP in the liver and lung compared to the group treated with PBS only (CLP). The enhanced infiltration of PMN into the lung and liver was also associated with additional histological aspects of organ injury, for example, steatosis and ballooning degeneration in the liver or interstitial edema in the lung, as shown in the representative histological sections 24 hours after CLP (Figure 8). A scoring of this histological damage shows more intense organ injury of the liver 24 hours after CLP with additional rhDNase treatment in comparison to the CLP group treated with PBS only (CLP: 1.6 ± 0.6 versus CLP+rhDNase: 2.2 ± 0.3; mean ± standard deviation, n = 6). Correspondingly, lung damage was more pronounced in mice receiving DNase treatment after CLP (CLP: 1.3 ± 0.6 versus CLP+rhDNase: 2.2 ± 0.5).

## Discussion

This animal study shows that application of rhDNase leads to a profound and sustainable reduction of NETs-mediated bactericidal activity *in vivo*. More importantly, we could show that administration of rhDNase in a murine model of polymicrobial sepsis results in an advanced sepsis progression with temporarily increased mortality prevalence, enhanced bacterial dissemination as well as elevated neutrophil counts in sepsis-related organs, more severe organ injury, and higher levels of IL-6 in the circulation. Our data underline the important role of NETs as an accurate antibacterial mechanism in the early phase of infection in polymicrobial sepsis *in vivo*.

After an infection, PMN are the body's first line of defense and kill bacteria by phagocytosis or by the production of NETs. In a recent sepsis model, more bacteria have been found in NETs than were phagocytized by PMN or macrophages *in vivo *[17]. The authors concluded that snaring bacteria in NETs might be more effective than phagocytosis particularly under flow conditions. In the serious sepsis model presented here, the highest release of NETs was detectable within the first 24 hours after CLP. *Ex vivo *ability of PMN to release NETs was present over the entire observation period demonstrating that during sepsis there is no loss of PMN-mediated NET production. Interestingly, peak levels of induced NET release in PMN could be observed 24 hours after CLP showing good correlation with the highest cf-DNA values measured *in vivo *at this time. This suggests a possible priming of the NET-formation in the acute phase of sepsis. However, the above mentioned observations have some limitations since we studied PMN isolated from the bone marrow which might be functionally different from the circulating populations. The detailed signaling mechanisms leading to the shift from phagocytosis to NET formation during sepsis are poorly understood. Several factors have been described as being involved in the induction of PMN to form NETs. Observations in PMN of chronic granulomatous disease (CGD) patients, which exhibit severe defects in NADPH oxidase and fail to produce reactive oxygen species (ROS), revealed that ROS might contribute to the formation of NETs [26]. Furthermore, beside singlet oxygen, the Raf-MEK-ERK pathway, which is activated in many cells during sepsis, has been shown to be involved in NETs formation by promoting PMN activation [26,27].

There is a growing body of evidence that the interaction of PMN and activated platelets is largely involved in sepsis and that activated platelets can mediate PMN to rapidly make NETs *in vivo *[11,17]. It is well established that platelets, which are primarily involved in homeostasis, are decreased in sepsis. Thrombocytopenia is associated with severity and mortality during sepsis [28]. Clark *et al*. recently showed in a sepsis model *in vivo *that NETs formation induced by activated platelets was particularly evident in the liver sinusoids and the capillaries of the lung. Platelets become activated by LPS through the Toll-like receptor (TLR)-4 receptor and bind to PMN, thus triggering NET formation within a few minutes [17]. The authors suggested that the platelets function as a barometer in the blood, becoming activated only under very serious systemic infections to stimulate PMN to release the proteolytic and chromatin material, thereby increasing the capacity of the innate immune system to trap and kill circulating bacteria. These findings are in line with our data, showing an inverse correlation between NETs levels and platelets 24 hours after CLP. In mice treated with rhDNase, a strong decrease in cf-DNA/NETs levels could be determined accompanied by a significant raise in the number of platelets. It might be assumed that the dissociation of NETs by rhDNase provokes a platelet mobilization from the bone marrow to compensate reduced NET amounts. Thus, thrombocytopenia during sepsis might represent a sequel of an adherence of activated platelets to PMN during NET-formation. However, 48 hours after CLP the correlation between NETs amount and platelet counts was completely different with both decreasing dramatically over a period of 24 hours. Because we found PMN to be able to release NETs after *ex vivo *stimulation with PMA at this time, one might speculate that 48 hours after CLP, mice fail to produce enough NETs for disease control due to a lack of activated platelets.

In our model, levels of cf-DNA, as part of the NETs, were significantly reduced by continuous rhDNase application. Although overall survival after CLP was not affected by rhDNase treatment, mortality prevalence was significantly lower 24 hours after CLP and median survival was reduced in mice treated with rhDNase. Thus, it appears that NETs production by activated PMN is a crucial antibacterial effector in the early phase of infection *in vivo*. This assumption is further supported by our data obtained six hours after CLP showing that, in contrast to the control group, rhDNase-treated mice displayed a rapid and considerable increase of CFU counts at the site of infection as well as in the lung. Nevertheless, 24 hours after CLP a rebound was detectable with fewer CFU counts in the blood in mice treated with rhDNase. It seems, therefore, that provoked reduction of the amount of NETs early after sepsis induction inevitably leads to a disturbed eradication of bacteria at the infection site allowing bacteria to spread in the system. However, in a preliminary experiment rhDNase treatment up to 72 hours after CLP did not influence the survival of the mice, arguing again for the superior role of NETs only during the early phase after infection. It is possible that the missing effect of rhDNase on the overall survival rate is explained by our experimental protocol. Here, we used a comparable serious bacterial load and an overall survival rate of 20 % after six days on average. In addition, the formation of the NETs was abolished by rhDNase treatment only within the first 48 hours after CLP.

Therapeutic administration of rhDNase is currently used to treat chronic pulmonary disease in cystic fibrosis and systemic lupus erythematosus [29]. There is evidence that treatment with rhDNase is associated with an improvement in lung function [12]. However, the influence of rhDNase treatment on bacterial colonization of the airways in cystic fibrosis has been investigated with conflicting results. While Frederisken *et al*. found rhDNase application to be beneficial to cystic fibrosis patients, in another more recent study the authors did not find any long-term effects of rhDNase on the pulmonary bacterial airway colonization [30,31]. However, the most profound difference was found for S*taphylococcus aureus *and gram negative bacteria. Recently, the ability of important gram positive bacterial pathogens such as *Streptococcus pneumoniae *and also certain *S. aureus *strains to resist NET-dependent killing has been linked to their ability to secrete nucleases, which might also explain such a change in bacterial findings after rhDNase therapy. However, in this study we did not further differentiate bacterial infiltration in detail. Although macroscopic examination of CFU plates did not reveal any significant differences (data not shown), rhDNase therapy in sepsis potentially might lead to a shift of bacterial colonization to bacterial strains with a certain resistance to NET-dependent killing *in vivo*, which could influence the value of bacterial dissemination into organs and the outcome. Nevertheless, this speculative assumption should be addressed in further investigations.

Most pathogens are killed after they have been trapped by NETs [12,16]. Pathogens entrapped in the NETs are dispatched possibly after being exposed to a high local concentration of antimicrobial proteins [12,32]. On the other hand, it has recently been shown, that microorganisms captured by NETs and thought to be killed remain viable in the construct by undefined mechanisms [33]. Therefore, it has been suggested that confinement of bacteria to the local site of infection and thus prevention of further spread of bacteria, is another important function of NETs. Having shown that maximal release of NETs is achieved within 24 hours after CLP, this confinement of bacteria by NETs is obviously temporally restricted and their relevance in the late phase of sepsis seems to decline. Moreover, depending on the amount of bacteria, the physical barrier built by NETs may break down in time and allow bacteria to disseminate into sepsis-related organs.

During early sepsis, PMN become activated and lodge primarily in the capillaries of the lungs and the sinusoids of the liver [34]. In our study, neutrophil counts were significantly increased 24 hours after CLP in the lung and liver of mice treated with rhDNase and more pronounced injury has been found in these organs. IL-6, an important proinflammatory cytokine in response to infection, is increased after CLP in mice [35]. Indeed, in our model, blood levels of IL-6 increased significantly, reaching maximal levels 24 hours after CLP. In accordance with our previous data suggesting aggravation of infection by rhDNase treatment, IL-6 levels were found to rise faster in the rhDNase-treated group, and the highest cytokine concentrations were measured after six hours. These results indicate that the elimination of NETs exacerbates the inflammatory process early after CLP resulting in a shift of the inflammatory response to an earlier time point.

## Conclusions

NETs formation plays an important role in bacterial control during polymicrobial sepsis *in vivo*. It becomes evident that depletion of NETs modulates the immune reaction and aggravates the pathology that follows a polymicrobial sepsis *in vivo*. Strategies of NETs induction rather than NETs destruction might be a promising therapeutic approach for septic patients in the future.

## Key messages

• Application of rhDNase leads to a pronounced and sustainable reduction of NETs-mediated bactericidal activity in a mouse model of polymicrobial sepsis (CLP) *in vivo*.

• Administration of rhDNase results in an advanced sepsis progression with increased mortality prevalence and enhanced bacterial dissemination *in vivo*.

• Administration of rhDNase does not alter overall survival after CLP.

• rhDNase therapy is associated with elevated neutrophil counts in sepsis-related organs, enhanced tissue damage and higher levels of IL-6 in the circulation early after CLP *in vivo*.

• Formation of NETs is an accurate antibacterial mechanism in the early phase of infection in polymicrobial sepsis *in vivo*.

## Abbreviations

ALT: alanine aminotransferase; anova: analysis of variance; cf,circulating free; CFU: colony-forming units; CLP: cecal ligation and puncture; DAPI: 4',6-diamidino-2-phenylindole; DNase: desoxyribonuclease; ELISA: enzyme-linked immunosorbent assay; FCS: fetal calf serum; FITC: fluorescein isothiocyanate; H & E: haematoxylin and eosin; IL: Interleukin; ip: intraperitoneal; NETs: neutrophil extracellular traps; PBS: phosphate-buffered saline; PMA: phorbol-12-myristate-13-acetate; PMN: neutrophils; rh: recombinant human.

## Competing interests

The authors declare that they have no competing interests.

## Authors' contributions

WM, TL and SF conceived the study, analyzed and interpreted the data and drafted the manuscript. AP-G interpreted the data, contributed to the writing of the manuscript, and critically revised the manuscript. Experimental work was performed by WM, AH, SB, and CM. IW and JW critically revised the manuscript for intellectual content and gave important advice. All authors read and approved the final manuscript.
